# Regulatory dynamics of arginine metabolism in *Staphylococcus aureus*

**DOI:** 10.1042/BST20240710

**Published:** 2024-12-10

**Authors:** Itidal Reslane, Gabrielle F. Watson, Luke D. Handke, Paul D. Fey

**Affiliations:** Department of Pathology, Microbiology, and Immunology, University of Nebraska Medical Center, Omaha, NE 68198, U.S.A.

**Keywords:** amino acids, arginine, auxotrophy, metabolism, *Staphylococcus aureus*

## Abstract

*Staphylococcus aureus* is a highly significant pathogen with several well studied and defined virulence factors. However, the metabolic pathways that are required to facilitate infection are not well described. Previous data have documented that *S. aureus* requires glucose catabolism during initial stages of infection. Therefore, certain nutrients whose biosynthetic pathway is under carbon catabolite repression and CcpA, including arginine, must be acquired from the host. However, even though *S. aureus* encodes pathways to synthesize arginine, biosynthesis of arginine is repressed even in the absence of glucose. Why is *S. aureus* a functional arginine auxotroph? This review discusses recently described regulatory mechanisms that are linked to repression of arginine biosynthesis using either proline or glutamate as substrates. In addition, recent studies are discussed that shed insight into the ultimate mechanisms linking arginine auxotrophy and infection persistence.

## Introduction

*Staphylococcus aureus* is a gram-positive bacterium that asymptomatically colonizes ∼30% of the population, primarily in the anterior nares [1,2]. Asymptomatic carriers are predisposed to infections by their colonizing strain, frequently manifesting as skin and soft tissue infections, including folliculitis, impetigo, cellulitis, and abscesses [3–7]. Additionally, due to its capacity for hematogenous dissemination, *S. aureus* is implicated in severe systemic infections, such as osteomyelitis, endocarditis, and necrotizing pneumonia [6,8].

As *S. aureus* can colonize or cause infection in virtually any niche of the human host, does it require the acquisition or biosynthesis of specific carbon or nitrogen sources within these various niches? What staphylococcal metabolic pathways are critical during a *S. aureus* infection? Most importantly, Richardson and colleagues have found that *S. aureus* depends upon glycolytic activity to initiate an infection in both bacteremia and skin and soft tissue models of infection [9,10]. Indeed, *S. aureus* encodes up to 11 carbohydrate transporters, 4 of which transport glucose [9]. Similarly, as proline biosynthesis is regulated by carbon catabolite repression and CcpA [11,12], proline acquisition is required during growth in niches where glucose is abundant [13]. Supporting this hypothesis, the inactivation of the two primary proline transporters ProT and PutP results in a 5 log_10_ reduction in bacterial burden in a murine model of bacteremia [13]. Furthermore, proline acquisition during chronic pulmonary infection fuels *S. aureus* oxidative metabolism via the TCA cycle, providing a competitive advantage for persistence in fibrotic airway environments [14].

The dependence of *S. aureus* upon glycolytic activity during infection is related to host nitric oxide (•NO), which binds to heme iron and therefore inhibits respiration [15]. To generate PMF, *S. aureus* must pump out hydrogen ions via the ATPase utilizing ATP, and in addition, facilitate redox by fermentation generating lactate using an •NO insensitive lactate dehydrogenase [16,17] Therefore, gluconeogenic activity is dispensable during the initial stages of an infection [10]. However, once an infection becomes established or persistent, *S. aureus* is encased within hypoxic environments where •NO is limiting thus allowing for respiration [9,10]. Moreover, the anoxic conditions induce HIF-1α within host immune cells therefore increasing glucose consumption [18,19] Within these glucose-depleted conditions, *S. aureus* can utilize alternative carbon sources such as lactate [20], free amino acids [21] and peptides [22]. However, within these persistent infection niches, free arginine becomes limiting due to expression of host arginase surrounding the abscess [23]. Therefore, one would presume that *S. aureus* would be selected to induce arginine biosynthesis in environments lacking free arginine. However, early staphylococcal research demonstrated that *S. aureus* is an arginine auxotroph, requiring arginine for growth [24–26]. This review discusses the proximate mechanisms that govern arginine biosynthesis repression and further discusses possible ultimate mechanisms linking arginine biosynthesis repression to staphylococcal biology.

## Arginine auxotrophy in *S. aureus*

**Figure 1.**
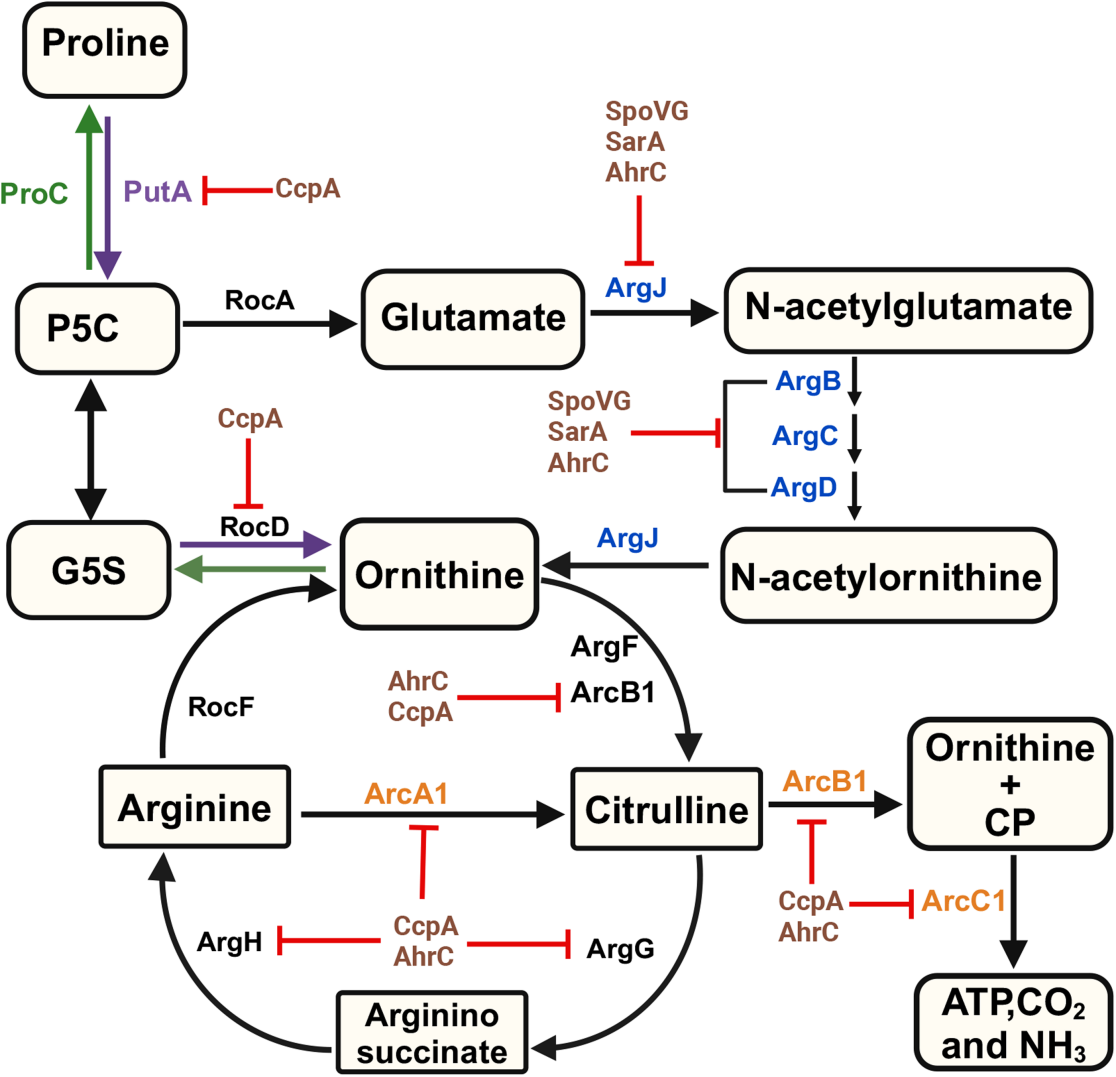
Regulation of arginine biosynthesis from proline and glutamate in *Staphylococcus aureus*. Glutamate is converted into *N*-acetylornithine by the enzymes encoded by the *argJBCD* operon, which is repressed by SpoVG, SarA, and AhrC. *N*-acetylornithine is then deacetylated by ArgJ to produce ornithine. Additionally, ornithine can be synthesized from proline via PutA and RocD (indicated by purple arrows), with the transcription of *rocD* and *putA* repressed by CcpA. Ornithine, a crucial intermediate, is subsequently converted into arginine by the enzymes ArcB1, ArgG, and ArgH. *arcB*1 and *argGH* are transcriptionally repressed by both AhrC and CcpA. Arginine is converted back into ornithine by RocF, which then serves as a substrate for proline biosynthesis via RocD and ProC (indicated by green arrows). In the arginine deiminase pathway (shown in orange), arginine is converted to citrulline by ArcA1, followed by its conversion into ornithine and carbamoyl phosphate (CP) by ArcB1. CP is then hydrolyzed by ArcC1 to generate ATP, CO_2_, and NH_3_. Moreover, glutamate can be synthesized from pyrroline-5-carboxylate (P5C) via RocA. The *arcA1B1D1C1* operon is repressed by CcpA and AhrC under aerobic conditions.

*Staphylococcus aureus* encodes pathways within the core genome that function to synthesize arginine either via the canonical or alternative pathways using glutamate or proline as substrates, respectively [27] (Figure 1). Despite encoding these biosynthetic pathways that synthesize arginine, *S. aureus* is an arginine auxotroph necessitating supplementation of arginine for robust growth in a chemically defined medium [21,23,26,28,29]. This auxotrophy may reflect an evolutionary adaptation to the arginine-rich environment of the nares, where *S. aureus* typically resides as a commensal [30,31]. The abundance of arginine in this niche may lead to a functional dependency, causing the repression of arginine biosynthesis and reliance on transport [30,31]. Supporting this hypothesis, human-colonizing staphylococcal species are generally auxotrophic for arginine, unlike environmental species that are prototrophic [25]. This suggests that human-adapted species like *S. aureus* repress biosynthesis due to high arginine concentrations in their colonization sites. Similar adaptations are seen in other bacteria such as *Escherichia coli* and *Pseudomonas aeruginosa*, where auxotrophy evolved in amino acid-rich environments [32–37]. In contrast with the above hypothesis, recent studies suggest that the inability to synthesize arginine in *S. aureus* enhances survival in acute infections where extracellular arginine is limited due to entry into a tolerant state via the stringent response [38]. Therefore, selection of arginine biosynthesis repression may be linked to ensuring that *S. aureus* enters a tolerant or persistent state during an acute infection where arginine is limited.

## Arginine biosynthetic pathways in *S. aureus*

### Canonical glutamate pathway

*Staphylococcus aureus* encodes the canonical glutamate pathway (*argDCJB*-*argF*-*argGH*) for arginine biosynthesis [27,39] (Figure 1), a conserved pathway across many bacteria including model organisms such as *Bacillus subtilis*, *Salmonella enterica* serotype Typhimurium, and *E. coli* [40–42]. This pathway involves eight enzymatic steps, using glutamate as a substrate and leading to the production of ornithine, which is subsequently converted to arginine [40–42] (Figure 1). The canonical pathway in *S. aureus* is characterized by very low transcriptional activity of the *argDCJB* operon across various growth conditions and repression of this pathway contributes to arginine auxotrophy in *S. aureus* [39,40,43]. Indeed, nuclear magnetic resonance studies in *S. aureus* JE2 USA300 revealed that arginine is not synthesized from C-labeled glutamate under standard growth conditions [21]. In our recent studies, we demonstrated that the *argDCJB* pathway is functional when expressed and that repression is mediated by three regulators including AhrC, SpoVG, and SarA [44]. The mechanism of regulation and the growth conditions used to investigate arginine biosynthesis using glutamate as a substrate will be elaborated further in this review.

### Proline catabolic pathway

A unique aspect of arginine biosynthesis in *S. aureus* is its ability to use proline as a substrate for arginine synthesis [23] (Figure 1). In this pathway, proline is first catabolized by proline dehydrogenase (PutA) to yield pyrroline-5-carboxylate (P5C), which is then spontaneously converted into glutamyl-5-semialdehyde (G5S) (Figure 1). G5S is subsequently converted by the enzyme RocD into ornithine, a key intermediate common to both the glutamate and proline pathways [21,23,28] (Figure 1). The reason *S. aureus* was selected to use this pathway is unclear, but one hypothesis is that *S. aureus* can access proline from host-derived proteins, such as collagen, which is degraded by staphylococcal proteases including ScpA and SspB [45–48]. Recent research supports this hypothesis by demonstrating that during chronic infection, *S. aureus* isolates up-regulate both ScpA, which cleaves collagen, and proline transporters ProT and PutP [14]. Transcriptional up-regulation enables *S. aureus* to degrade host collagen, releasing proline that is subsequently imported and metabolized [14]. Indeed, genes responsible for synthesizing arginine from proline such as *putA* and *argH* were up-regulated in *S. aureus* strains associated with chronic pulmonary infection [14]. Although unclear, these data may suggest that the metabolism of collagen-derived proline provides a substrate for arginine biosynthesis in chronic infections. In summary, the coexistence of the proline and the canonical glutamate pathways resulting in arginine biosynthesis in *S. aureus* suggests an evolutionary adaptation that enhances its metabolic flexibility. This dual pathway system likely confers a selective advantage by enabling *S. aureus* to thrive across diverse host environments and ecological niches, thereby supporting persistent colonization and infection.

## Regulation of arginine biosynthesis

### CcpA represses proline-dependent arginine biosynthesis

Carbon catabolite protein A (CcpA) mediates carbon catabolite repression thus repressing secondary carbon source catabolic pathways (including amino acid metabolic pathways) and enabling *S. aureus* to utilize preferred carbon sources such as glucose [49–51]. In *S. aureus* JE2, growth in a complete defined medium containing glucose (CDMG) is robust but is completely inhibited when arginine is removed [21,23]. However, growth in CDMG lacking arginine (CDMG-R) is rescued in a *ccpA* mutant [23] due to derepression and thus increased expression of *argGH*, *arcB1*, and *putA* which facilitates arginine biosynthesis using proline as a substrate [23]. Note that USA300 *S. aureus* strains, currently one of the most prevalent strain backgrounds causing infection in the United States, has two arginine deiminase operons (native and ACME encoded ADI operons) and thus two ADI encoded ornithine carbamoyl transferases (OCT) [27,52]. To differentiate between the two and for the purposes of this review, we have annotated the native OCT ArcB1 and the ACME encoded OCT ArcB2.

### AhrC represses arginine biosynthesis using proline as a substrate

The ArgR-type regulators including ArgR/AhrC are conserved across bacterial species. *S. aureus* USA300 isolates (or those that encode the ACME pathogenicity island) encode three homologs of ArgR/AhrC including ArgR1 [27,39], ArgR2 [27,52], and AhrC [27,39]. Based on studies performed in model organisms, these regulators function in an arginine-dependent manner repressing arginine biosynthetic genes in the presence of arginine [53–60]. This regulation involves an N-terminal DNA-binding domain and a C-terminal arginine-binding domain that mediates arginine sensing [57–59,61–63]. In the absence of arginine, the affinity of ArgR/AhrC for DNA decreases, resulting in increased arginine biosynthetic gene expression and subsequent growth [57–59,61–63]. However, in *S. aureus*, the removal of arginine does not alleviate the repression exerted by AhrC [28] indicating that the transcriptional repressor AhrC remains active without its corepressor [28]. This sustained repression may result from mutations in operator sites that enhance DNA binding affinity or from amino acid substitutions in AhrC that strengthen its interaction with DNA independent of arginine. The latter mechanism was documented in the *E. coli* B strain lineage where a single amino acid substitution allows ArgR, an AhrC orthologue, to remain active without arginine, diverging from the regulatory pattern seen in *E. coli* K-12 [64]. However, no such mutations have been identified in *S*. *aureus* AhrC to account for the continuous repression. It is possible that AhrC possesses intrinsic properties enabling it to bind DNA tightly and mediate repression regardless of arginine presence. Further electrophoretic mobility shift assays and/or isothermal titration calorimetry studies are required to determine if AhrC binds to the *argGH/arc* promoter(s) at similar affinities in the presence or absence of arginine.

### AhrC, SpoVG and SarA transcriptionally regulate arginine biosynthesis using glutamate as a substrate

Our previous research indicated that the *argDCJB* operon, responsible for arginine biosynthesis using glutamate as a substrate, is only modestly (∼5-fold) up-regulated in an *ahrC* mutant [28], contrary to reports suggesting that the inactivation of AhrC should significantly enhance *argDCJB* transcription [57,59,60]. However, although the *argDCJB* is slightly up-regulated in an *ahrC* mutant, arginine is biosynthesized using proline as a substrate, not glutamate [28]. This led us to hypothesize either the involvement of additional regulatory mechanisms or a non-functional *argDCJB* operon. However, subsequent studies demonstrated that ectopic overexpression of the *argDCJB* operon enables growth in the absence of arginine indicating its functionality [29,44]. Further investigation revealed that arginine biosynthesis using glutamate as a substrate is active in a *S. aureus* JE2 *spoVG sarA* mutant highlighting the function of SpoVG and SarA in repressing the *argDCJB* operon [44]. Interestingly, RT-PCR showed that AhrC senses the presence of arginine and represses *argDCJB* even in the presence of *spoVG* and *sarA* mutation demonstrating that AhrC is a key regulator of this operon [44]. This indicates that the subtle change in expression of *argD* in the *ahrC* mutant [28] was due to repression by SpoVG and SarA and not the lack of AhrC repression of *argDCJB* [44]. Although *argDCJB* is repressed by both SarA and SpoVG and the operon is not fully depressed in an *ahrC* allelic replacement mutant, we propose that AhrC acts as an arginine sensor, relieving subtle repression of the *argDCJB* operon when arginine is absent. It is presently unclear how AhrC acts as an arginine sensor in the context of *argDCJB* operon repression, yet constitutively represses other genes, such as *argGH* or *arcABDC*, irrespective of arginine concentration. Furthermore, the mechanism of interplay between SarA and SpoVG in the regulation of *argDCJB* requires further study.

### *S. aureus* selects for mutations to grow in the absence of arginine

Based on previous work in other bacterial species, we hypothesized that *S. aureus* would be able to grow in defined medium lacking both glucose and arginine (CDM-R). The hypothesis is based on the premise that, without glucose, CcpA repression is lifted [21,23], and arginine depletion triggers AhrC dissociation from DNA [57,59,62]. This allows the transcription of arginine biosynthetic genes leading to the production of arginine [41,57]. Surprisingly, *S. aureus* JE2 displayed an extended lag phase and a reduced growth rate in CDM-R [28]. However, compensatory mutations, including one in *ahrC* and another in the arginine deiminase upstream regulatory region, restored robust growth, phenocopying the wild-type strain in CDM [28]. This reliance on mutation-driven activation rather than conventional derepression is also observed with other amino acids such as valine where *S. aureus* selects for mutations in *codY* to grow in a defined medium lacking valine [65]. The mutations in these regulators provided a valuable model to study the unique mechanism of amino acid biosynthesis regulation in *S. aureus*. In particular, the selected mutations in CDM-R allowed us to investigate arginine biosynthetic regulation under different growth conditions [21,23,28,44], providing a deeper understanding of *S. aureus* physiology and its adaptive strategies in challenging environments.

In the first class of mutants, genome sequencing documented that mutations in *ahrC* facilitated the growth of JE2 WT in CDM-R. RT-PCR analysis revealed that the *ahrC* mutation led to a significant up-regulation of *argGH* and *arcB1* expression, enabling arginine biosynthesis via proline as noted in a *ccpA* mutant. Complementation of *ahrC* in the *ΔahrC* background abrogated growth in CDM-R, confirming that AhrC actively represses arginine biosynthesis in the absence of arginine. Additionally, a *ccpA* mutation also facilitated growth in CDM-R suggesting that CcpA represses arginine biosynthesis independent of glucose availability. Interestingly, *ahrC* transcript levels were unchanged in the *ccpA* mutant, while *argGH* expression was significantly up-regulated. These findings indicate that both AhrC and CcpA act cooperatively to repress arginine biosynthesis in CDM-R as CcpA does not function to regulate *ahrC* transcription [28].

Secondly, single nucleotide polymorphisms (SNPs) supporting growth in CDM-R were identified upstream of the ATG start site of the *arcA1* gene, which is part of the native arginine deiminase operon [28]. These SNPs, termed P*arc* mutants, are unique because they are located upstream of the proposed AhrC and CcpA operator binding sites, within untranslated regions rich in A/T repeats, rather than within the operator sites themselves [28]. P*arc* mutants phenocopy the growth of the Δ*ahrC* mutant in CDM-R but differ transcriptionally [28]. Distinctly, P*arc* mutants show no increase in *argGH* transcription and only a slight increase in *arcB1* transcription during growth in CDM, whereas both genes are significantly up-regulated in CDM-R [28]. This finding suggests that AhrC remains active in P*arc* mutants repressing *argGH,* and to a lesser degree *arcB1,* in the presence of arginine. However, in the absence of arginine, AhrC-dependent repression of *arcB1* is alleviated facilitating arginine biosynthesis and growth.

## *arcB1* enzymatic activity is essential for *S. aureus* arginine biosynthesis

Both classes of mutants exhibited enhanced expression of *arcB1* and *argGH* which mediated growth in CDM-R [28]. Induction of these genes using cadmium inducible promoter was performed to explore if the transcriptional repression of *argGH* or *arcB1* contributes to the block of arginine biosynthesis in CDM-R [28]. Overexpression studies confirmed that increasing the expression of *arcB1* but not *argH* restored the growth of *S. aureus* in CDM-R indicating that the conversion of ornithine into citrulline is the critical enzymatic step in the biosynthesis of arginine [28] (Figure 1). In addition to mutation-driven growth, ornithine supplementation also supports JE2 growth in CDM-R by increasing the transcription of the catabolic OCT *arcB1* but not the anabolic OCT enzyme encoded by *argF* [28]. It is currently unclear how ornithine induces *arcB1* transcription. The ability of ArcB1 to restore growth in the presence of ornithine is unexpected given its catabolic function of converting citrulline into ornithine [66–69]. However, our data document that the conversion of ornithine to citrulline, an anabolic reaction, is catalyzed by ArcB1 [28]. We hypothesize that *S. aureus* is selected to utilize ArcB1, a catabolic and thus inefficient anabolic OCT, to sustain arginine auxotrophy within the population [28]. Therefore, based on our data, in the absence of arginine, *arcB1* transcription is significantly reduced thus repressing arginine biosynthesis. However, if mutations (*ahrC/*P*arc*) are selected increasing ArcB1 concentration or the intracellular concentration of ornithine (substrate) is elevated, arginine biosynthesis proceeds. These observations indicate that *S. aureus* employs a combination of transcriptional and substrate-level (ornithine) induction to tightly regulate arginine biosynthesis.

## The impact of elevated P5C concentration on *S. aureus* growth in CDM-R

P5C is an intermediate of both arginine and proline biosynthesis [11,12,21,70,71] (Figure 1). To synthesize proline, P5C is reduced to proline through the activity of P5C reductase, ProC [11,12,21,70,71] (Figure 1). Notably, mutations in *rocA* and *proC* presumably increase the intracellular P5C pool by limiting its conversion to glutamate and proline, respectively [28,29]. This proposed redirection of P5C toward arginine biosynthesis and intracellular ornithine concentration restores *S. aureus* growth in CDM-R [28,29]. Supporting these observations, overexpression of PutA supports growth in a medium lacking arginine by enhancing proline-to-P5C conversion [29]. We propose that *S. aureus* prioritizes the P5C pool for proline or glutamate biosynthesis, potentially due to a higher affinity of ProC/RocA for P5C as compared with RocD, which functions to generate ornithine from P5C. Indeed, maintaining a high intracellular concentration of proline is essential, as limiting proline transport significantly reduces bacterial burden during infections [13]. In conclusion, the preferential use of P5C for proline or glutamate biosynthesis over ornithine and subsequent arginine biosynthesis may be a key mechanism by which *S. aureus* limits arginine biosynthesis and promotes auxotrophy.

## Host-induced selective pressure and arginine biosynthesis

*Staphylococcus aureus* bloodstream infections followed by dissemination to distal organs expose the bacteria to selective pressures including host immune responses and nutrient limitation [72,73]. Following intravenous inoculation in mice, *S. aureus* migrates from the liver to the kidneys, where it establishes chronic infections characterized by renal abscesses [72,74–76]. During hepatic transit, a bottleneck effect occurs resulting in genetic drift and reduced genetic diversity as evidenced by clonal expansion [72]. Notably, the bacteria initiating renal abscesses do not harbor mutations that enhance fitness; rather, lesion formation appears stochastic with random cell selection [72]. Instead, the shift in environmental conditions at secondary sites of infection, such as the kidneys, promotes the selection of *S. aureus* variants capable of establishing chronic infection [77–79]. Adaptive evolution in this context is demonstrated by the increased frequency of beneficial mutations in key regulatory genes such as *agr*, *walKR*, *rsp*, and *yjbH* [77–81] as well as in metabolic genes such as *sucA*-*sucB* and antibiotic resistance loci [80,82,83].

**Figure 2.**
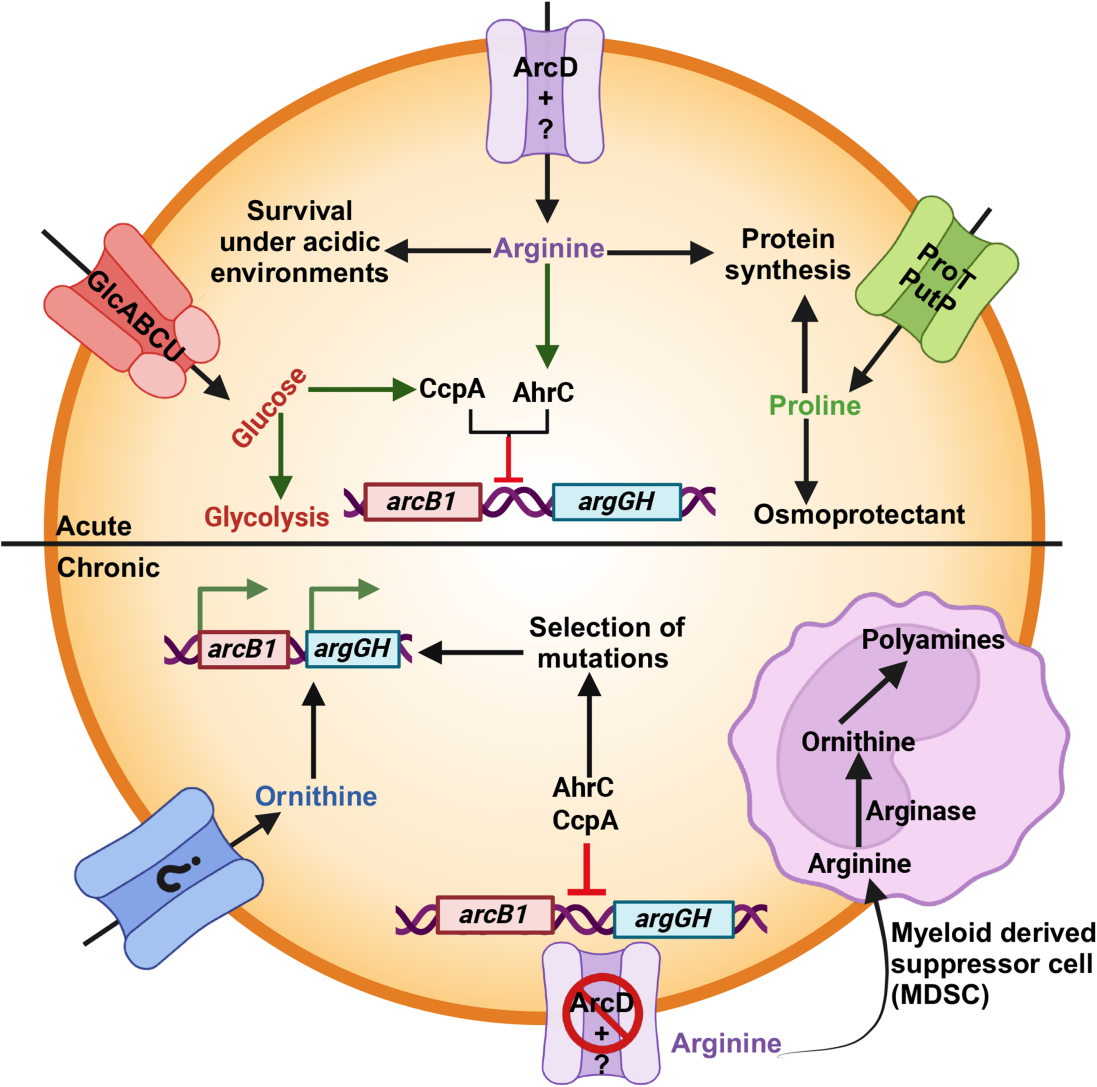
Arginine availability and adaptive response of *Staphylococcus aureus* in a murine model of infection. In the acute phase of infection (upper part of figure, labeled acute), glucose is transported by multiple carbohydrate transporters, promoting growth and energy production via glycolysis. Proline, a key osmoprotectant, is acquired through the transporters ProT and PutP, which are critical for survival in glucose-rich environments. Arginine is transported into the bacterial cell through ArcD1 and additional, yet unidentified, transporters. During this stage, arginine biosynthesis is repressed by the regulatory proteins AhrC and CcpA, preventing the activation of biosynthetic pathways when extracellular arginine is available. As the infection progresses to the chronic phase (lower part of figure, labeled chronic), particularly within nutrient-limited environments such as renal abscesses, myeloid-derived suppressor cells (MDSCs) deplete extracellular arginine by converting it into ornithine and polyamines via arginase activity. Despite the depletion of arginine, *S. aureus* maintains repression of the *argGH* biosynthetic operon by AhrC and CcpA. To overcome this repression and adapt to arginine-limited conditions, *S. aureus* selects for mutations that alleviate repression and activate arginine biosynthesis. In addition to mutation-driven derepression, we propose that ornithine may be imported into the bacterial cell, where it enters the urea cycle and induces ArcB1 activity, contributing to de novo arginine biosynthesis. Whether ornithine is supplied via host arginase activity or scavenged from the extracellular environment remains to be investigated. Created with Biorender.com.

During persistent infections as modeled in the kidney, *S. aureus* encounters a microenvironment rich in myeloid-derived suppressor cells (MDSCs), which deplete extracellular arginine by converting it to ornithine and polyamines via arginase activity [84–89] (Figure 2). We surmise that *S. aureus* exploits the host's arginase response not only to evade NO-mediated killing but also to adapt to the nutrient-limited conditions within abscesses. Furthermore, the conversion of arginine to ornithine by MDSC arginase provides *S. aureus* with a precursor for *de novo* arginine biosynthesis (Figure 2). This ability to utilize ornithine can be particularly advantageous since it can bypass AhrC repression by enzymatically inducing ArcB1 activity, a key enzyme in the arginine biosynthetic pathway, as described above.

Notably, early in infection, arginine auxotrophs are prevalent in all organ systems including the kidneys; however, by day 20, arginine prototrophs emerge as the dominant population in the kidneys using mouse models of infection (unpublished data). These prototrophs, which are absent in the early stages of infection, carry mutations, among others, in the *ahrC* gene and the regulatory region upstream of the *arcA1B1D1C1* operon (unpublished data), enhancing their ability to synthesize arginine as previously noted (Figure 2). Interestingly, ∼50% of *S. aureus* clinical isolates can grow in defined medium lacking arginine and glucose further highlighting their relevance and suggesting that arginine biosynthesis is selected in certain host niches [28]. Taken together, the selective emergence of prototrophs at the chronic stage suggests that these mutations are specifically advantageous in the kidney environment, highlighting the function of adaptive evolution in *S. aureus* persistence and survival in nutrient-limited conditions during chronic infection.

## Conclusion

The metabolic versatility of *S. aureus* highlights its capacity to proliferate and persist in diverse and hostile environments. The sophisticated regulatory mechanisms governing arginine biosynthesis, influenced by both genetic and environmental factors, are potential examples of the evolutionary adaptation of *S. aureus* to the human host. By elucidating the functions of key regulators such as AhrC, SpoVG, and SarA, and examining the impact of adaptive mutations, we have gained significant insights into the metabolic plasticity that drives *S. aureus* virulence and the biological function that underlies repression of arginine biosynthesis even in the absence of arginine. Investigating the biological relevance linking the regulation of certain metabolic regulatory pathways and their function within the natural niche enhances our understanding of the metabolic intricacies of this adaptable pathogen and enables the development of strategies to mitigate its risks and reduce its impact on human health.

## Perspectives

- Understanding the *S. aureus* metabolic pathways that facilitate infection will provide new understanding of how this pathogen causes disease and may provide new targets for antibacterial development.
- Arginine biosynthesis using either proline or glutamate is heavily repressed by multiple regulators including CcpA, AhrC, SpoVG, and SarA.
- New data suggests that arginine auxotrophy may be linked to ensuring that *S. aureus* enters a persistent or tolerant state when free arginine is depleted in the host, such as what is encountered during infection. However, this is in contrast with data documenting that mutations are selected during infection which facilitate arginine biosynthesis. Perhaps arginine biosynthesis is selected against during acute infection whereas mutations are selected facilitating arginine biosynthesis during persistent infections as modeled by a 20 day murine kidney infection. Further studies will provide other ultimate mechanisms linking arginine and amino acid auxotrophy in bacterial pathogens.

## Abbreviations

MDSC: myeloid-derived suppressor cell
OCT: ornithine carbamoyl transferase
P5C: pyrroline-5-carboxylate
SNP: single nucleotide polymorphism
